# Hunting and consumption of rodents by children in the Lassa fever endemic area of Faranah, Guinea

**DOI:** 10.1371/journal.pntd.0009212

**Published:** 2021-03-17

**Authors:** Moussa Douno, Emmanuel Asampong, N’Faly Magassouba, Elisabeth Fichet-Calvet, Marí Sáez Almudena

**Affiliations:** 1 Projet des Fièvres Hémorragiques en Guinée, Centre de Recherche en Virologie, Université de Conakry, Guinée; 2 Department of Social and Behavioral Sciences, School of Public Health, University of Ghana, Legon, Accra, Ghana; 3 Department of Virology, Bernhard Nocht Institute for Tropical Medicine, Hamburg, Germany; 4 Center for International Health Protection, Robert Koch Institute, Berlin, Germany; School of Veterinary Medicine University of California Davis, UNITED STATES

## Abstract

As a consequence of the Ebola outbreak, human–animal contact has gained importance for zoonotic transmission surveillance. In Faranah (Upper Guinea), daily life is intertwined with rodents, such as the Natal multimammate mouse, *Mastomys natalensis;* a reservoir for Lassa virus (LASV). However, this contact is rarely perceived as a health risk by residents, although Lassa fever (LF) is known to be endemic to this region. Conversely, these observations remain a great concern for global health agendas. Drawing on ethnographic research involving interviews, focus group discussions, participant observations, and informal discussions over four months, we first identified factors that motivated children to hunt and consume rodents in Faranah villages, and thereafter, explored the knowledge of LF infection in children and their parents. Furthermore, we studied two dimensions of human-rodent encounters: 1) space-time of interaction and 2) factors that allowed the interaction to occur and their materiality. This approach allowed us to contextualize child-rodent contacts beyond domestic limits in the fallow fields, swamps, and at other times for this practice. A close look at these encounters provided information on rodent trapping, killing, and manipulation of cooking techniques and the risk these activities posed for the primary transmission of LASV. This research facilitated the understanding of children’s exposure to *M*. *natalensis* during hunting sessions and the importance of rodent hunting, which is a part of their boyish identity in rural areas. Determination of when, where, why, and how children, rodents, and environments interacted allowed us to understand the exposures and risks important for human and animal surveillance programs in the Lassa-endemic region.

## Author summary

Hunting and animal handling by children are considered important aspects of zoonosis occurrence. Children are implicated as the high-risk group for Lassa spillover. In a context where public health interventions pay little attention to the role of children in the occurrence of zoonotic infections, we conducted a qualitative study to explore factors that motivate children to hunt and eat rodents and to understand the practices that may constitute pathways of Lassa virus primary transmission in the endemic area of Faranah, Upper-Guinea. Moreover, we sought to extend the anthropological analysis of human–animal interactions to children who hunt, as researchers are gaining interest in exploring their hunting practices for the origins of zoonotic diseases. Our findings highlight the sociocultural, economic, and environmental dimensions of childhood and generate policies and interventions on the roles children can play in the primary transmission of zoonotic diseases to humans. These insights may aid in planning prevention programs for zoonotic infections in Lassa fever-endemic areas.

## Introduction

Animal and human health are interdependent, with pathogens crossing over between species, sometimes leading to disease. Zoonotic agents are an increasing threat to global health. At least 60% of all human pathogens are of zoonotic origin [1], and mammals are the main reservoir hosts for most of the known zoonotic diseases in humans [2,3]. Specifically, several species of rodents are known to harbor pathogens, including viruses, such as Lassa virus (LASV) that cause approximately 66 zoonoses in humans. Some of these rodents are qualified as “hyper reservoirs,” as they can carry 2–11 zoonoses [4]. The consumption of bushmeat, especially rodent meat, has been related to improved food security in rural communities [5]. Sociocultural, economic, and environmental factors play a major role in facilitating encounters between humans and these mammals, thereby resulting in the emergence of zoonosis [6]. These factors imply that anthropological analyses are key components in a study on zoonotic infection pathways [7]. Anthropology brings attention to the social existence and interaction between humans and animals in daily life [8–10].

Among the zoonotic diseases, Lassa fever (LF) is a threat to the West African population [11,12], affecting approximately 300,000 people and causing 5,000–10,000 deaths annually in the region [13]. The disease is caused by the LASV, which is transmitted to humans through contact with an infected neonatal multimammate mouse, *Mastomys natalensis*; the principal rodent reservoir of the virus [14]. The main route of primary transmission is by the contamination of food or household items with rodent urine or feces [13,14]. Moreover, poor housing is a factor that offers rodents an opportunity to easily aggregate in dwellings in rural settings. Human behaviors that bring residents of the rural areas into contact with rodents have been reported in certain endemic countries. For instance, in Nigeria (where frequent epidemics occur), Sierra Leone, and Guinea, rodent hunting and consumption have been identified as risk factors for the primary transmission of LASV [15,16].

Rural communities alongside the tropical forests of West and Central Africa rely on bushmeat as a nutrititive, economic, and cultural component of their livelihoods [5,17]. Wild animal hunting and butchering constitute the major risk of primary transmission of zoonotic diseases because of human exposure to animal body fluids. The probability of infection is determined by the frequency and nature of the contact residents have with animal hosts [18]. For instance, hunting and butchering promoted the occurrence of outbreaks of monkeypox [19] and Ebola virus disease (EVD) [20,21], infections with simian foamy virus [22], and primate T-cell lymphotropic viruses [23]. Engagement of children in hunting of small mammals such as rodents, has been documented in West Africa, where LF is endemic [16,24,25].

Rodent hunting and consumption is an ancient human activity in the world; Peruvians have been eating guinea pigs for centuries [26,27]. Rodents constitute a substantial portion of the wild animals consumed by humans worldwide [26]. Generally, they are not protected by game laws, are usually abundant near dense human populations, and are easy to catch [16,25]. Furthermore, they are described as important predictors of food security compared with other taxa [5]; a study in Nigeria reported that they were the most hunted prey [28].

Recently, studies identifying risk factors for zoonosis and emerging pathogens have focused on a better understanding of human-rodent interactions worldwide [4,29]. One risk factor for the transmission of infectious diseases is rodent hunting and consumption due to the intimate contact with animal body fluids during butchering. However, rodent hunting is important considering its contribution to children’s diet. A 2015 study in Lao PDR, Asia, demonstrated that contact with rodents through hunting, preparing, and eating was common among different ethnic groups, ages, and genders [29].

Rodent hunting has been depicted as the activity of children, especially boys [16,24,25]. Moreover, rodent hunting and consumption by children in Guinea have been previously documented [9,25,30]. Previous studies have mentioned hunting as one of the household responsibilities of children as they are sometimes sent to search for meat for the whole family [31–34]. Hunting is considered one of the population’s subsistence strategies; thus, children learn this practice through informal peer-imitation, observation, and play [32]. The learning process requires little or no direct training by adults [31]. Hence, in LF endemic regions, children, mainly boys aged ≥ 6 years, are exposed to the risk of LASV primary transmission through direct contact with rodents. Indeed, the abundance and migration of *M*. *natalensis* between houses and proximal cultivation fields, where children go to hunt, have been reported in Upper-Guinea [12,15,35]. Although children’s involvement in hunting, handling, and consumption of rodents and other wild animals has been reported in similar regions in sub-Saharan Africa [24,36,37], the social aspects of the role played by these children are often underestimated by public health interventions.

This study aimed to explore factors influencing rodent hunting and consumption by children and understand the exposure parameters responsible for LF infection in this endemic area of Faranah, Guinea. Our study provides new insights into the investigation of zoonotic disease pathways by unveiling the valuable sociocultural, economic, and environmental factors for children’s exposure to LASV reservoir in this region. The findings facilitate contextualization of human-animal encounters by demonstrating that children may play a major role in LASV primary transmission to humans. These findings would aid in defining public health interventions, especially community-based strategies for behavioral change through communicative mechanisms; such strategies will enable the people in these villages to understand the risk incurred by this young population, and develop strategies to prevent and protect them from this risk.

## Methods

### Ethics statement

The study protocol was reviewed and approved by the Guinean National Ethics Committee for Health Research (No. 027/CNERS/19). The participants were informed regarding the research in Malinke. The study information sheet was read out and explained to the participants and the parents or guardians of the children. Participants’ right to decline and withdraw from participation at any time was emphasized. Written consent was obtained from all participants and their witnesses prior to data collection. Parents or guardians of children consequently signed the written consent form.

### Study site and population

Faranah prefecture in the savanna region of Upper Guinea, located 452 km from Conakry, served as our study site. Administratively, it is divided into 11 sub-prefectures and one urban area and is essentially an agro-pastoral zone, with a predominantly rural population of approximately 299,612 individuals [38]. The origins of the main ethnic groups in the villages are Malinke and Djallonke. These groups are traditionally hunters and farmers and are predominantly Muslims. Most of them are illiterates and live below the poverty line [38], and their main activity is agriculture. Farming is organized in the fields surrounding the villages. Each family has its own territory for cultivation.

#### Rodent control and LF in Upper Guinea

**Fig 1:**
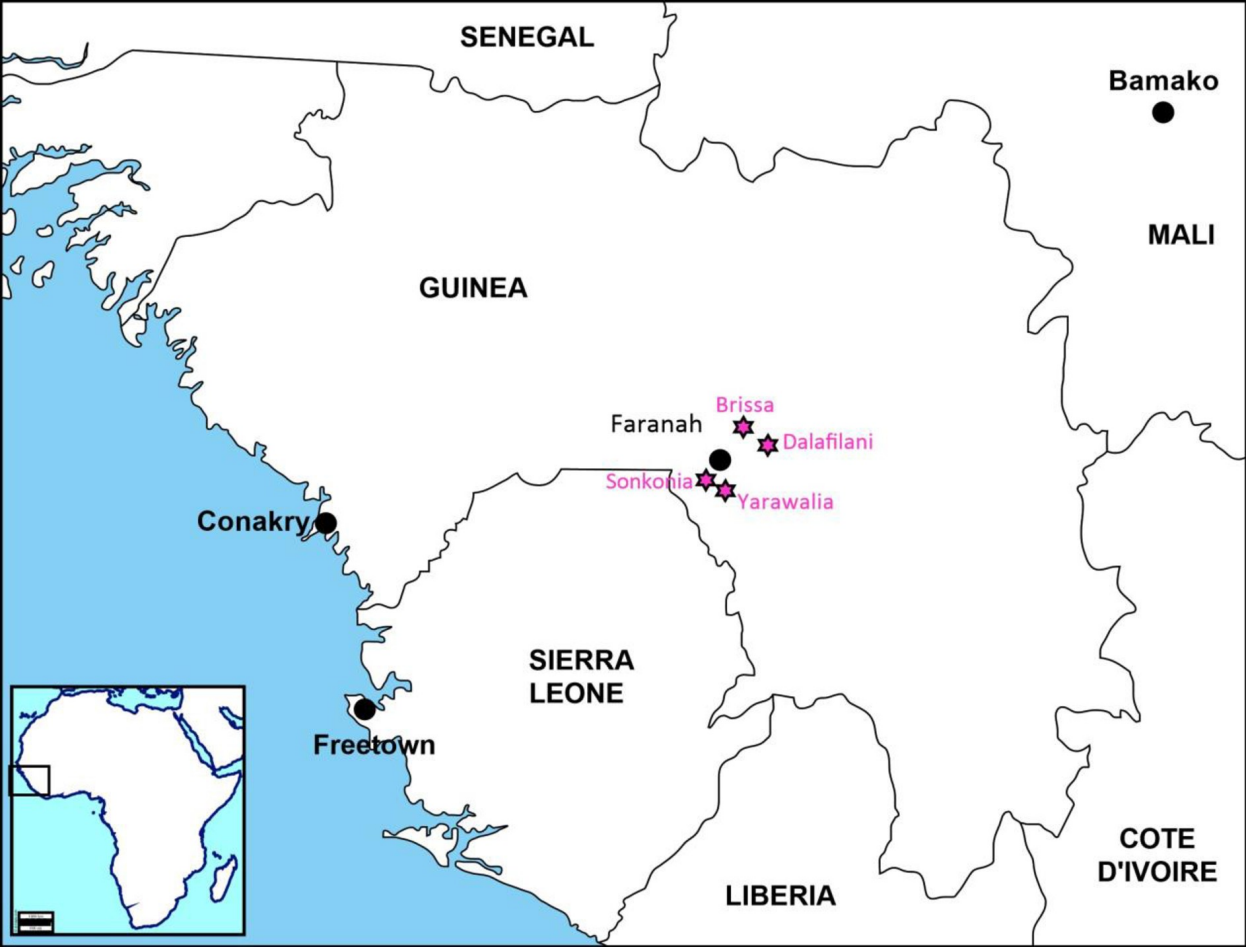
Location of the four villages in the surroundings of Faranah prefecture, Guinea.

Faranah prefecture is known as an endemic area for LF in the Upper Guinea [11,12,15,39–41], and the presence of LASV in *M*. *natalensis* was mainly found in animals caught in houses and proximal cultivation fields [15,35]. A multidisciplinary and international consortium funded by the Deutsche Forschungsgemeinschaft, titled “Lassa fever in Guinea and Sierra Leone: rodent control and seasonality of human exposure to Rodents (LAROCS),” investigated the human seroprevalence, rodent control, and anthropological aspects of human-rodent interactions in the Faranah prefecture from 2013 to 2019. The LAROCS project was conducted in six villages selected based on the LASV prevalence in rodents (range: 2%– 21% [40]) in Faranah to assess the feasibility, acceptability, and effectiveness of rodent control intervention. In the absence of a vaccine, rodent control and human behavior are the main strategies for controlling LF. Previous sociocultural findings have also been published [8,9,16,40,42]. Based on the emergent hypothesis from a previous study on the role of children in hunting, the study was conducted in four of the six villages—Brissa, Dalafilani, Sonkonia, and Yarawalia (Fig 1). Villages were visited daily or at least five times per week, and sleeping over was avoided because of the risk of Lassa infection. Data were collected for four months (from February to March 2018 and from December 2018 to January 2019).

### Observations

Eight observations were conducted before, during, and after hunting sessions. These covered diverse activities, such as hunting preparation, leadership, division of roles and actions, digging, storage and transport of dead rodents, butchering, cooking/roasting, and consumption. Special attention was paid to hunting sites, rodent species, hunting techniques, and rodent handling practices from capture to consumption, considering intimate contact with animal body fluids.

Initially, groups of children and adolescents who hunt rodents were identified. Thereafter, their parents were informed regarding the study goal, and permission to participate in their children’s hunting sessions was obtained from them. In swamps, hunting gatherings included boys aged 6 to 12 years and hunting dogs, whereas in bush, hunting gatherings included boys aged 13 to 16 years, male adolescents aged ≥ 17 years, and dogs. One hunting session involved adolescents and some adults had hunting guns. All the hunting sessions took place in the daytime and lasted for approximately 3–5 hours. Girls were excluded from these expeditions because of gender-based division of labor and identity, with hunting being a male activity. The activities were documented immediately after returning from the fields as memos in a Word file. Moreover, photographs were captured during the hunting sessions.

### Informal discussions and interviews

We conducted informal discussions with community members (children, young adults, and adults of both sexes). In this process, we identified individuals to conduct semi-structured interviews. This process was conducted until data saturation was attained. Discussions were conducted in Malinke, the common native language spoken in these villages. Discussions were based on their interactions with rodents beyond the domestic space, especially the practice of rodent hunting by children and their knowledge regarding LF. The information was immediately elaborated in a Word file (memos) to document these activities during fieldwork. A total of 25 in-depth interviews (IDIs) of adults (men and women) and nine focus group discussions (FGDs) (five with children, one with young male adults, and three with adults [two with women and one with men]) were conducted in the four villages. The children were 6–16 years old, some were schooled, and others were unschooled. The young adults were 21–24 year–old males and included only single farmers. The adults were 35–78 years old and included farmers, hunters, village leaders, elders, and housewives. Two FGDs and five IDIs were conducted in each village, except in Dalafilani, where an additional FGD and four additional IDIs were conducted and in Sonkonia, where an additional IDI was conducted. The reasons for this inequality were as follows: a) the first author was trained in ethnographic practice in Dalafilani and Sonkonia villages, and b) theoretical sampling was performed. Most often, men were occupied with farm work (harvesting and preparing new cultivation fields); hence, they were rarely present in villages within the timeframe of our study. Interviews were recorded with an audio recorder and lasted for 30–80 minutes.

### Data processing and analysis

Audio recordings were transcribed verbatim from Malinke to French first, and thereafter, to English. Furthermore, the field notes were translated into English. All transcripts and field notes were cross-checked for consistency. Data gathered were rendered anonymous and stored on the personal password-protected computer of the researcher with limited access to the research team.

The coding process began by importing all transcripts into the QSR International NVivo 12 Plus software for analysis. A codebook was created based on the objectives of the study, each transcript was opened in NVivo software, and all statements were coded line-by-line into nodes. The coding was reviewed, and some nodes were rearranged, whereas others merged to develop themes and sub-themes. Throughout the coding process, the initially developed codebook was revised and refined. Thematic analysis was performed using both deductive and inductive processes [43].

## Results

### Hunting context

#### Habitat

The Faranah region is characterized by a mixture of savanna and forest, which includes the National Park of Upper Niger where wildlife is becoming rare. Different habitats, such as swamps, agricultural lands, old cultivation fields or fallow lands, and forests, were identified along with the participants. Swamps are flooded areas. Uncultivated swamps are found in deep forests far from the villages, probably explaining why they were not cultivated. Cultivated swamps are found near the villages and close to water sources. Hence, they are conducive for cultivating rice and potato leaves. After harvesting the rice, grasses grow in the swamps until the next planting season and cows feed on these grasses. Hence, until the next rice planting, dense herbs, cane forests, and few trees grow in the swamps. In general, agricultural lands are cleared for farming. Men cut the trees in an area and prepare the land for the cultivation of crops, such as rice, cassava, and groundnuts when a family wants to start a new farm. Agricultural lands are used on a rotating crop basis, starting with rice, groundnuts, and cassava before fallowing the land. Old cultivation fields or fallow lands are recognizable by the mounds that are made to grow cassava and the trees had not reinvaded the space. Forests or wooded savannas include tall trees and reeds, sparse areas, and traces of fire in places with fallen trees. Trees found in the forest are *Ceiba pentandra* (silk cotton tree) and *Tectona grandis* (teak). Food trees, such as *Parkia biglobosa* (African locust bean), *Combretum micranthum* (Quinqueliba), *Adansonia digitata* (Baobab), *Vitellaria paradoxa* (shea tree), *Saba senegalensis* (Madd fruits), and *D*. *senegalense* (Tallow tree) are present along some fruit trees, such as *Persea americana* (avocado), *Mangifera indica* (mango), *Citrus X sinensis* (orange), and *Citrus X limon* (lemon).

#### Rodent species

**Fig 2:**
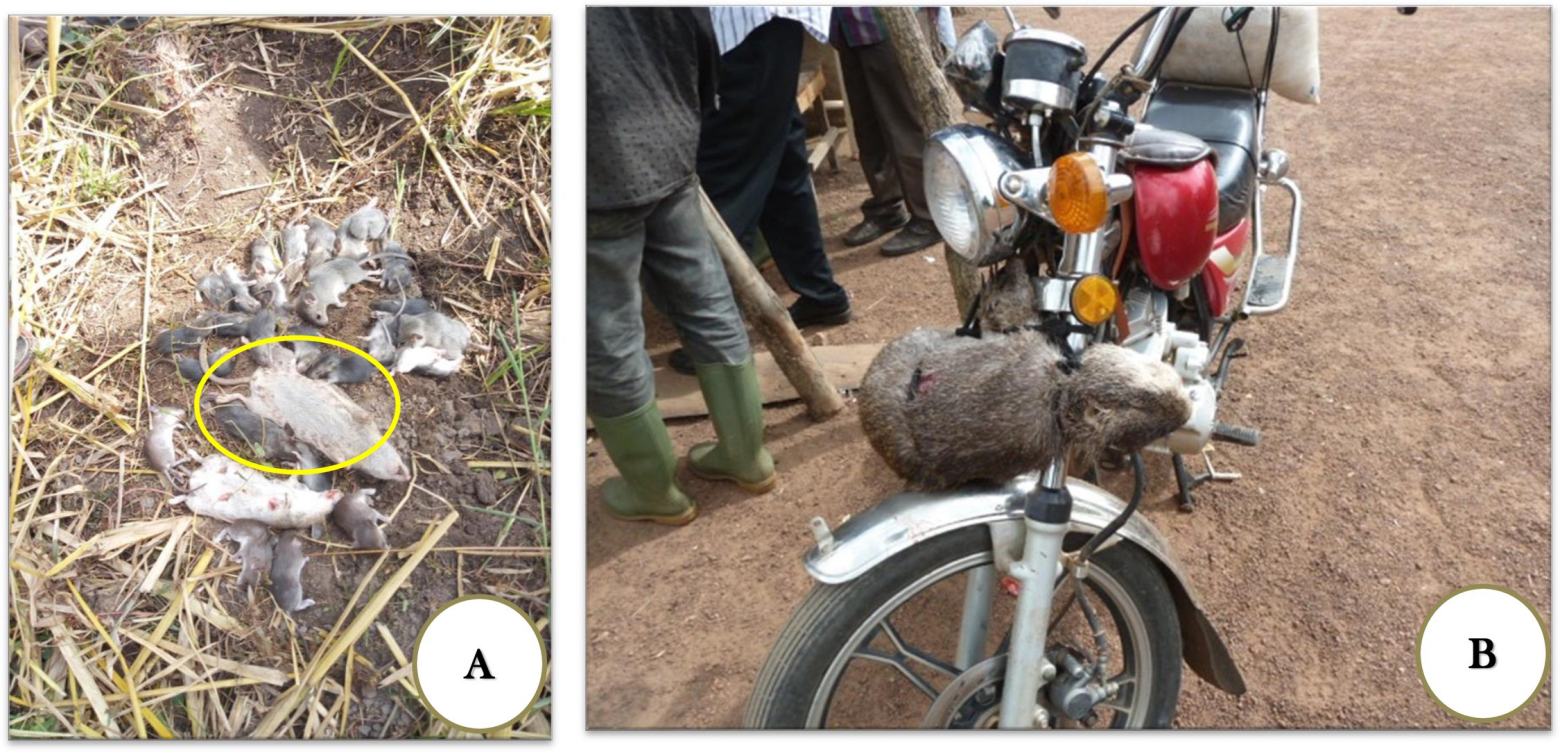
Hunting trophies (A) Small-bodied rodents including the multimammate mice (*Mastomys spp*) caught by children aged 6–12 in swamps. (B) A large-bodied rodent (*Thronomys swinderianus*) caught by adults in cane bush.

In this study, mice and rodents were referred to as small mammals that children and adolescents could hunt in the study villages. Different species were differentiated based on the size, color, and hairiness of the animal and were not collectively termed in the local language. Rodents were grouped into two categories based on their weight (see Table 1 for size specification) as follows:

- Small-bodied rodents, collectively termed “souris” in French, “mice” in English, and “gnina” in Malinke, are found in houses, swamps, and forests [44]. Among them, the following species were identified by the participants and cross-checked by a rodent ecologist using photos: *Mastomys spp*. (Fig 2A), *Arvicanthis sp*., *Uranomys ruddi*, and *Praomys daltoni*. *Mastomys spp*. (locally termed *faragbön*) such as *M*. *natalensis* and *M*. *erythroleucus*.

**Table 1:** Categories of rodents based on their weight.

Vernacular name	Scientific name	Weight	Place	Age of children	Techniques
**Farah gbön**	*Mastomys spp* (Fig 2A)	40 g	Swamps	6 to 12 years	Burn rice stalks to see burrows, dig burrows with hoes, use sticks and/or step on mice to kill them, and catch mice with bare hands
**Kassan gnina**	*Arvicanthis sp*.	120 g	Swamps		
**Gnina gbenin or Soumbou Gninanin**	*Uranomys ruddi* *Praomys daltoni*	30 g 30 g	Swamps Old cultivations		
**Torowah**	*Cricetomys gambianus*	1 kg	Old cultivation fields	13 to 16 years	Dig burrows on the termite mounds or on the floor with hoes, set fire in the burrows, and use sticks to push the animal out
**Kèrèn**	*Xerus erythropus*	0.5 kg	Old cultivation fields		
**Kögnina**	*Thryonomys swinderianus* (Fig 2B)	4 kg	Cane bush (reeds), uncultivated swamps, forest	>17 years	Set fire to the bush/cane, chase the animal with dogs, and kill the animal with cutlass or sticks, or shoot the animal with a gun

Large-bodied rodents that were identified were as follows: giant rat (*Cricetomys gambianus*), squirrel (*Xerus erythropus*), marsh cane rat (*Thryonomys swinderianus* (Fig 2B)), and hare (*Lepus victoriae*). Giant rats (locally termed *torowah*) were more often found in termite mounds in forests.

### Socialization of children

In Malinke and Djallonke communities, children are educated in the family. They begin practicing religious and social norms, such as Muslim prayers and basic activities, such as feeding cows, helping in farms, and household chores from the age of 7 years. At this time, gender-based separation and division of labor is noted considering the types of game, use of time, movement inside and outside villages, and tasks. For instance, boys play or walk in the village or fields by themselves, whereas girls are usually asked to be at or nearby home and help in domestic tasks. Boys follow their fathers to the farms to help with farm work (plowing, fencing, planting cassava, caring for cows, and scattering birds) from the age of 7 years, whereas girls stay with their mothers and help with domestic chores, such as cooking, fetching water, cleaning, and caring for their younger siblings. Young girls help their mothers in groundnut and rice fields and gardens when the mothers go to farms. Furthermore, boys engage in hunting expeditions, and girls are left behind, but may be invited by their brothers, other relatives, or friends to share their prey and cook for them.

### Hunting initiation

In these villages, hunting is a part of the boys’ daily lives. Rodent hunting by children is emphasized by adults as a common practice (E1 in S1 Table), and considered to be a traditional and customary practice continued through generations. One of them referred to this practice as “*generational culture*,” meaning that boys gradually get involved in age-based hunting practices and learn the techniques by imitating their elders (E2 in S1 Table). Small children start rodent hunting in cultivated swamps from the age of 7 years to acquire techniques, such as digging, staying attentive, jumping on animals, and killing animals with bare hands. Gradually, children learn and acquire the strength necessary to hunt large-bodied rodents in other habitats. During hunting expeditions, children may find wildlife animals, such as monkeys, tortoises, and varans that they carry to the village to sell or eat.

### Hunting technique

The main hunting technique involves digging burrows. Children leave the village with a game bag, machetes, hoes, and slingshots. They use these tools and bare hands during hunting. They identify a burrow and put their hands into it in search of rodent hair to detect their presence before digging. If a rodent is present, they dig alone or in turns until the rodent comes out. Alternatively, they may use sticks and/or put fire on the thatch and introduce it into the burrow to asphyxiate giant rats in their shelter and push them to come out. However, if they feel heat in a burrow, they move away from the burrow and continue walking, considering that a snake may be present (E3 in S1 Table).

Another technique employed is the use of hunting dogs. Hunting is observed to be a group task in which skills are exercised, techniques are learned, and complementary roles are performed by the group members, including dogs. Hunting dogs aid children in different ways. First, the dogs detect burrows with their sense of smell, dig the burrow along with children, stay alert for movements, and are ready to intercept any rodent leaving the hole. Second, the excitement of a dog indicates the presence of an animal in the burrow, which encourages children to continue digging until the animal comes out and is caught either by the dog or by the children themselves (E4 in S1 Table). Finally, the dogs protect children as they are capable of detecting the presence of a snake in a burrow with their sense of smell, and thus, alert the children by fleeing from the burrow (E5 in S1 Table). A team spirit is seen in the way the prey is shared among the members; the dogs usually get the head.

### Selection of hunting locations

Hunting sites are selected based on seasonality, age and physical characteristics of participants (capacity of digging and strength for walking long distances), and accessibility to rodent habitat. Rodent hunting and consumption occur mainly during the dry season. During agricultural tasks, children go for rodent hunting on the farm, with the argument that they protect family crops that can be destroyed by these rodents. This is emphasized by the adults who explain that the protection of cultivation fields was the first goal of rodent hunting, with a great focus on marsh cane rats, which destroy most of the rice fields.

However, when children are free of family chores during the day, they decide to go for hunting. In swamps, the ground is wet and burrows are not deep; hence, it is easier to dig. Smaller children go for hunting mice in cultivated swamps after harvest, which is the favorable period, as mice enter rice stalks in the quest of food. In old cultivation fields and forests, the ground is dry and burrows are hard and deep, making it difficult to dig. Uncultivated swamps are the favorable shelters of marsh cane rats, and hence, stronger teenagers and adults, including hunters, go there for hunting. Finally, in old cultivation fields, older children target giant rats, squirrels, and hares in the termite mounds and burrows.

**Fig 3:**
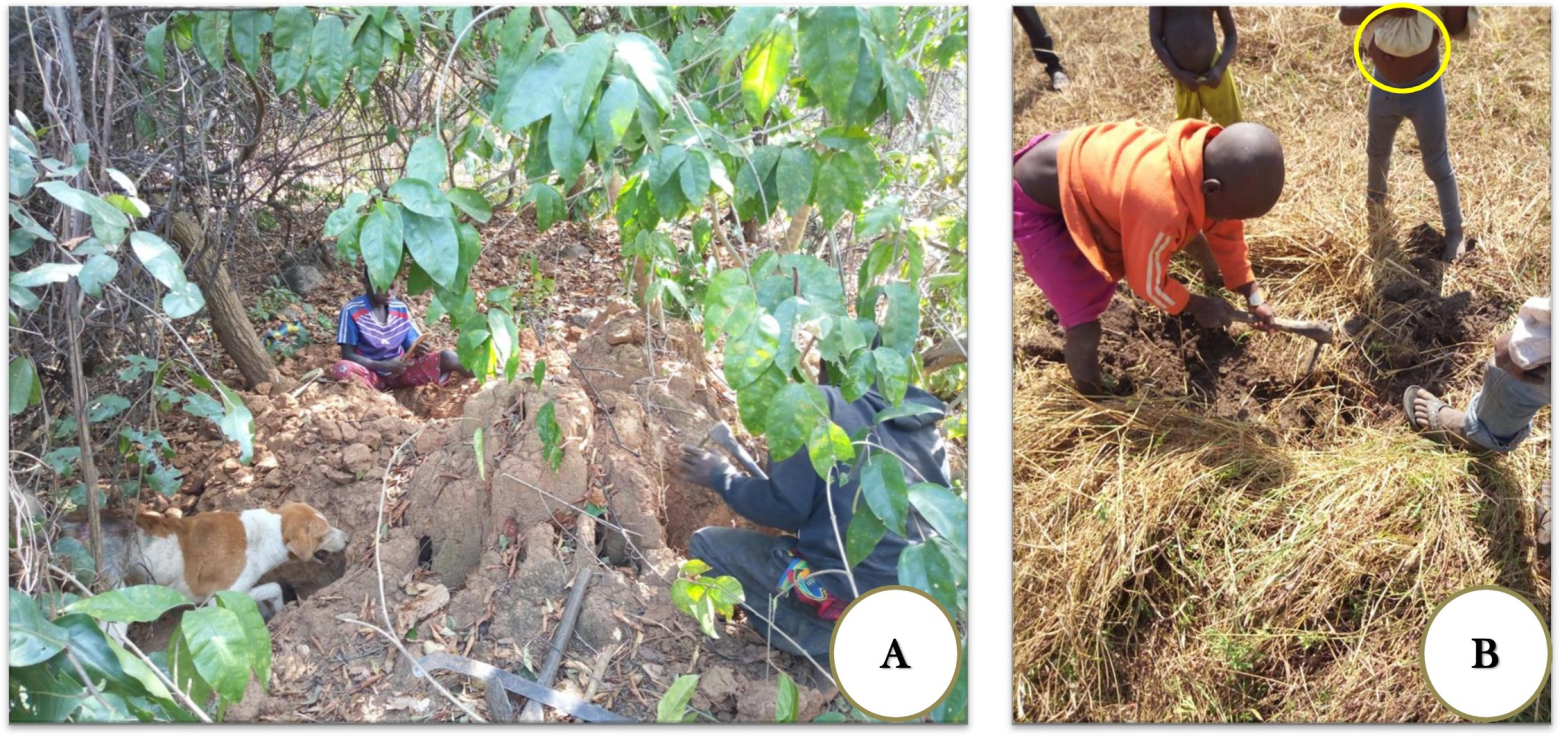
Hunting techniques (A) Older children accompanied by a dog, digging rat burrows on a termite mound in the bush. (B) Smaller children digging mice burrows with a hoe in a swamp close to the village, with one of them holding the catch in his shirt.

Older and stronger children prefer hunting large-bodied rodents so they can give some meat to their parents and sell some to earn money. However, they occasionally hunt mice in swamps when no prey is found from hunting in the forest or when it is late to go to the forest for hunting large-bodied rodents. School boys are often concerned with the latter option because they leave school by 5 PM. Hence, they go to nearby swamps to catch and eat mice in the evening (Fig 3).

### Rodent handling in sharing and cooking practices

**Fig 4:**
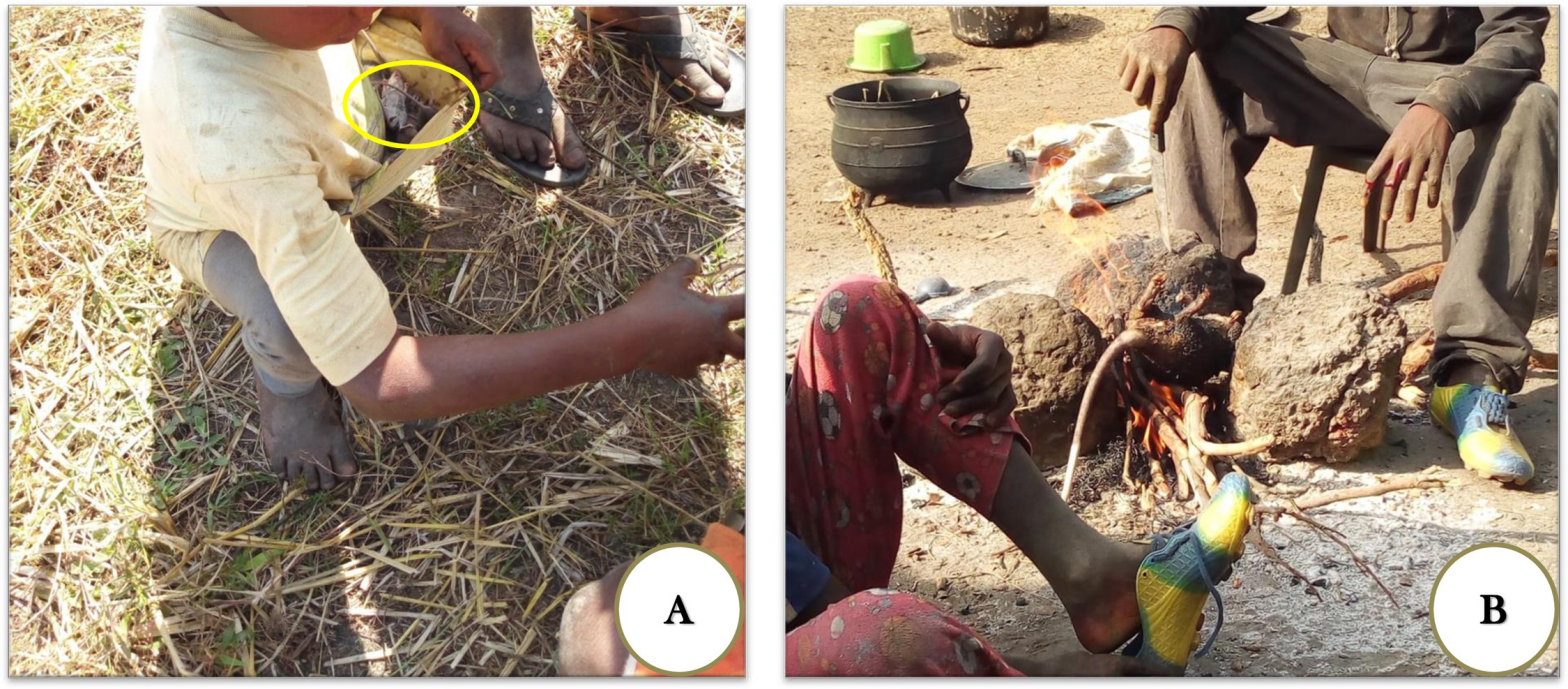
Collection and consumption (A) A boy collecting mice caught in a swamp to carry them home. (B) The boys singeing a rat (*Cricetomys gambianus*) at home after a hunting session in the bush.

After children hunt their prey, the next step is to carry it home, cut, cook, and eat. Hence, mice are gathered and put in pockets, shirts, or game bags by the youngest child of the group (Fig 4A). At home, all mice are gathered in one place, and the older child shares them among all the group members. Everyone takes their share to their home (E6 in S1 Table). Sharing is based on age, with the oldest children getting the bigger mice and the youngest getting the smallest. At home, they keep the dead mice in their rooms until evening to roast/grill them. This is because during the Harmattan, it is forbidden to light fire in the daytime to avoid fire outbreaks in the village, as almost all the houses have thatched roofs. During this time, the younger brothers and sisters are given the opportunity to play with these dead mice.

In the evening, children together with their siblings singe the animal hair, eviscerate, and grill or roast the mice over fire to eat. If they catch many mice, they buy or ask their mothers for oil (E7 in S1 Table) to grill mice with it in a cooking pot on their own after singeing and eviscerating to eat with friends (Fig 4B). However, with large-bodied rodents (rats in most cases), children may either share the meat after singeing and butchering the prey or cook and eat together with friends and younger siblings.

### Factors influencing hunting

#### Difficulty in accessing meat: Scarcity and poverty

It has been unanimously reported that meat is rare in the daily meals of people in these villages. Wildlife hunting and trading are limited to adult hunters. Meat from professional or opportunistic hunters is rare and most often not consumed at home because they often prefer to sell in Faranah for profit making. To prevent this, hunters are forbidden by local authorities (district chiefs) to carry the meat to town and are mandated to sell it in the villages. Generally, this rule is not followed, as some hunters occasionally hide and take their prey to Faranah for sale.

The general understanding is that children are involved in hunting rodents to fill in the lack of daily meat. Some children resort to dead domestic animals, such as chickens, which they grill with friends and eat (E8 in S1 Table). According to all respondents, another reason is that the giant rat is the preferred animal among all rodents captured by children for sale. Rodent hunting constitutes a source of income for children in these villages. The reason for selling giant rats is that its meat is considered an effective treatment for high blood pressure (E9 in S1 Table).

Dry or fresh fish are the main sources of protein. During the dry season, some women fish in streams close to the villages. In most cases, these fish and the meat bought from the market are not sufficient for the entire family. Hence, adults often share with their children during the family meal. This is based on the belief that a protein should be offered to adults as a symbol of respect: *“a child just eats rice*, *not protein (miyafén)*, *which is for adults”* or *“a child just asks to fill his stomach*, *but should not ask for protein*.*”* However, children have their own meat—mice and rat meat are considered to be “*children’s meat*.” If adults are seen eating rodents, people laugh at them, although rats are often given to mothers to cook the sauce for the family because of the lack of meat. Generally, meat, most often from domestic animals, such as cows and sheep, is consumed only during religious or family celebrations.

Moreover, poverty and the non-existence of butcher shops in the villages were mentioned as the main causes of meat scarcity in family meals. Poverty was particularly emphasized as the key factor that prevented parents from buying meat for their children (E10 in S1 Table). This situation encourages some parents to tell their older children who are capable of hunting large rodents to hunt for meat for the family.

#### Childish stubbornness, boyish identity, and intergenerational relationships

Rodent hunting was found to be an everlasting phenomenon during childhood (E11 in S1 Table), and adult males perpetuate hunting practices at large by buying and using guns for big game. Nevertheless, some parents ban their children from hunting because they are concerned for their security in bush. However, if children hunt against the will of adults, adults consider it to be childish stubbornness. This was reiterated by some boys who stated that their parents banned them from hunting, but they still practiced it. Children decide to go against their parents’ mandates because they think that going to the bush for hunting is something expected from them as young adults (E12 in S1 Table).

Additionally, children consider the bush as a good place to do everything that is forbidden in the presence of their parents. Hence, they go to such a place without adult supervision to do what they want. Unlike village, a bush is a place without forbiddances (E13 in S1 Table).

Hunting is a central experience for boys. In the villages, it is through hunting that children identify themselves as brave. It is a gender construction of what a male child should do. Children who practice rodent hunting have a particular perception of those who do not and see them as “*fake and lazy boys*.” After hunting, they refuse to share their prey with children who do not hunt (E14 in S1 Table), reinforcing the intra-gender rule, which is a shared thought of what boys, young adults, and adults should do.

**Fig 5:**
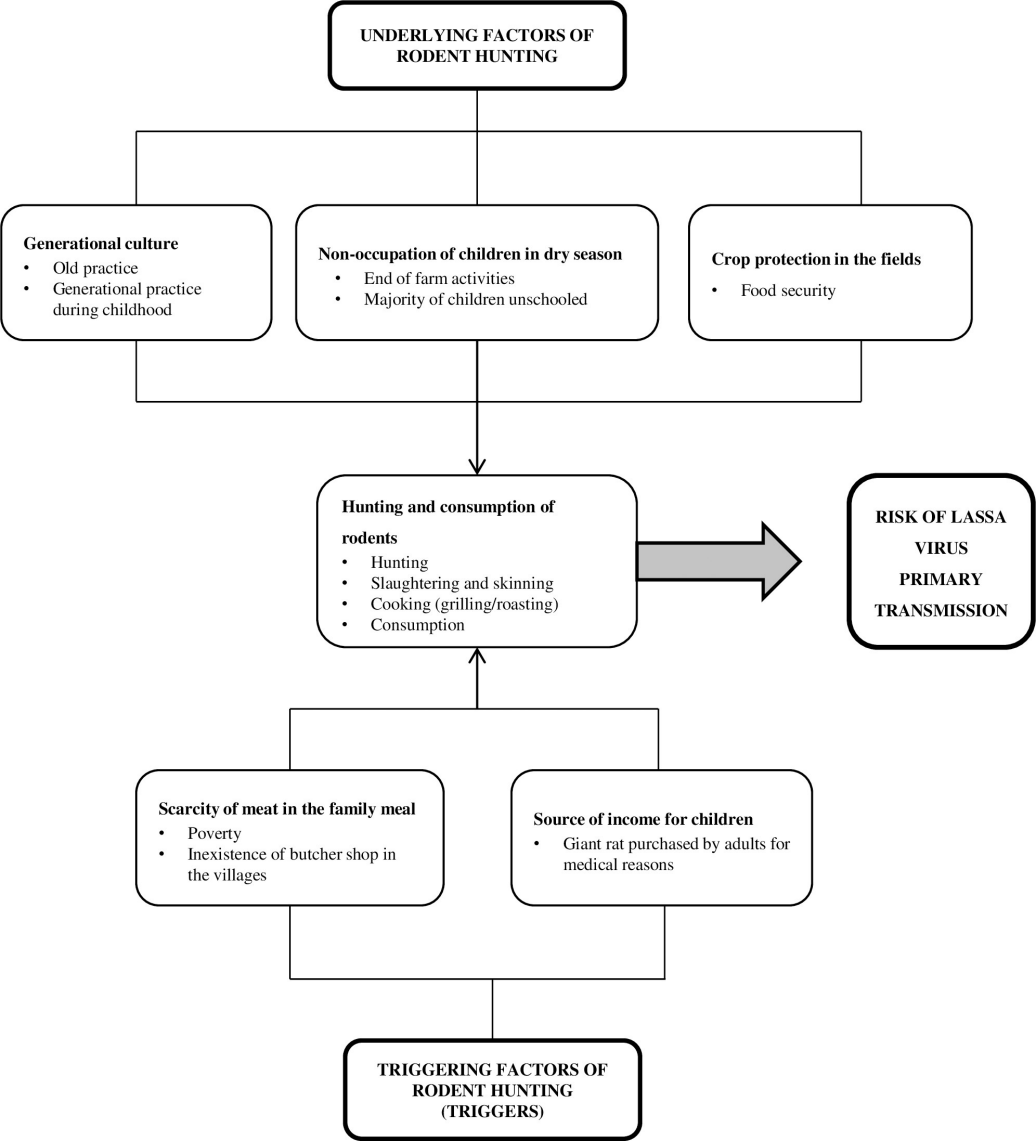
Factors influencing rodent hunting and consumption by children in the study villages.

These factors influencing rodent hunting by children are summarized as underlying and triggering factors in Fig 5.

### Knowledge of LF and perception of risk

#### Knowledge of LF and its vector

This study revealed that most of the adults and all the children respondents had no knowledge of LF. Respondents (both adults and children) referred to the 2014–2016 EVD outbreak in the country when asked about LF. In particular, children easily recalled this event because they were forbidden to hunt and eat rodents and other wild animals during that period (E15 in S1 Table).

However, few adults reported that they had heard of LF from the LAROCS workers who came for mice control and other research activities in their houses. They referred to the project workers as “*mice catchers*” and the disease as “*mice disease*.” Some of them were able to explain some symptoms of the disease (E16 in S1 Table), whereas others confused it with malaria (E17 in S1 Table).

Regarding the LF vector, many adults revealed that they were informed by the project workers that mice could transmit the disease to humans. However, they did not know the nature of the disease transmitted to people by mice (E18 in S1 Table).

Hence, based on this information regarding the human health risk due to mice, people in these villages believe that only mice in the houses are not good to be consumed because they have seen mice enter latrines and graves in cemeteries and feed on anything, thereby associating them with dirt and infections. Conversely, the bush mice are considered commensal meat, and children continue to hunt and consume them. No one sees them as a health risk for LASV transmission.

Although mice are associated with dirt and infections in the houses, children do not deliberately kill them to control pests. However, if they found a mouse in a position that allowed them to kill, they would kill it and throw it away. Pest control is the responsibility of adults who sometimes use poison or indomethacin to eradicate mice from the house.

#### Perceived risk of rodent hunting-related LF

Based on the aforementioned hunting techniques and handling practices (from catching mice to preparing them for consumption), children come into direct contact with mouse body fluids, such as urine, saliva, and blood. However, none of the respondents in this study mentioned the risk of LF primary transmission through rodent hunting practices of children. Instead, snakebite, injury, and tiredness were cited and perceived by both adult and child respondents as risks and threats to health during hunting sessions (E19 in S1 Table). In particular, adult respondents emphasize snakebite as their major concern during the rodent hunting expeditions of children because they had witnessed (E20 in S1 Table) or had personal experience about it (E21 in S1 Table).

## Discussion

### Meaning of rodent hunting for boys in rural Upper-Guinea

Our study revealed how the role of hunting is attributed to males in these villages. Moreover, this study revealed that the learning process begins early in childhood (age: approximately 6 years) and the practice is perpetuated throughout adolescence and adulthood [32,33]. The learning process constitutes the initiation phase, where they learn about bush and animals, as they are expected to contribute to meat searching for the entire family during adolescence [31–33]. Our study underlines the social importance of hunting for boys, as it shows that being able to hunt is a symbol of being a “*real man*.” Consequently, boys who did not hunt continuously experience peer pressure, and their intergenerational relations with those who hunted plays a major role in their involvement in rodent hunting practices. Similar findings showing the social importance of wild meat for boys in rural Sierra Leone were reported by Bonwitt et al. in 2017 [24].

Rodent hunting is the main means for meat procurement by children in these areas to compensate for the lack of protein in the family meal. Rodents are called “*childrens’ meat*.” Meat-sharing norms vary across Africa [24,44]. In our study, we found that protein (meat and fish) in the family meal is under the total control of adults who share and decide whether to give some to children. Additionally, this was underlined in a study by Bonwitt et al. in Sierra Leone in 2017 [24]. As a symbol of respect for adults, children should not be interested in protein unless adults give them. Hence, to have meat under their control, children learn to hunt. Moreover, they have complete control over the sharing of mice meat because it is not consumed by adults. This may be one of the key motivators of rodent hunting for boys in this area.

Our results suggest that despite protein intake for food security [5], children may be at a greater risk of zoonotic diseases, such as LF because they are motivated beyond than food as reasons for hunting.

Finally, poverty is the driver of rodent meat consumption. Upper Guinea is one of the poorest regions in Guinea [45]. Poverty is palpable in terms of living conditions, mainly habitat structure and the quality of building materials (mostly round thatched huts), and food intake (quality and quantity), leading to undernourishment of children [46–48], especially with regard to protein consumption in this area. Wildlife consumption tends to be higher in poorer households [49] because they cannot afford alternatives, such as cow meat sold in the city.

### Children-mice encounters and risk of LASV primary transmission

In hunting fields, children come into direct contact with mouse body fluids, especially urine, which is released by *M*. *natalensis*, the main vector of LASV, when it is frightened [37]. Repeated direct and voluntary contact occurs during transportation of mouse in pockets, shirts, or game bags to home, playing with dead mice, and meat preparation and consumption [13,33]. In this study, children mostly caught *Mastomys spp*. in cultivated swamps. This implies that because of potential regular contact with the LASV reservoir during hunting sessions, smaller children aged 6–12 years should be considered a high-risk group for LF. Furthermore, older and schoolchildren aged 13–16 years who occasionally hunt in the same swamps after school can be at risk for LF. As previously reported in similar areas [9,49], boys and girls (mainly their sisters invited for cooking) are opportunistically exposed to rodent bites, feces, and body fluids during meat preparation at home. The main division and importance of exposure distribution is repeated–intentional for boys, and opportunistic and unintentional for girls.

This indicates that children are in contact with the LASV reservoir beyond the domestic limits, where previous human-rodent contact has already been reported in these localities. *M*. *natalensis* is widely found in houses and proximal cultivation fields surrounding the villages [15]. Additionally, their migration between houses and surrounding fields has been documented [35]. Moreover, the LASV prevalence in *M*. *natalensis* has been determined in Brissa, Dalafilani, Sonkonia, and Yarawalia, ranging from 15% to 23% of animals infected in 2013–2015 [40,50,51]. Moreover, immunoglobulin G (IgG) prevalence in humans was found to be 84% (n = 1302 persons) in the Faranah area, with 62% of 6-year-old children being seropositive [52]. These new insights unveil the role that children may play in the primary transmission of LASV to humans through hunting practices in this endemic area. Previous studies performed in 1992 and 2004 have reported high seroprevalence (approximately 40%) in these rural settings [11,39].

In addition, the role played by domestic animals in LASV transmission should be further investigated. This is because in our study, dogs were members of hunting explorations and were in direct contact with the body fluids of rodents [53–55].

### Risk extension to family members

Children extend and increase the risk for the rest of the family members in two ways: first, they bring the mice caught in swamps back home and keep them in their rooms until cooking time, as reported previously. Moreover, they expose their younger siblings who play with these dead mice [9]. Second, they use cooking utensils (knives, cooking pots, and ladles) that are used by other family members to prepare the meat. Hence, all materials touched during meat preparation are contaminated by the biological fluids of the dead mice. These utensils may be used by their mothers again to cook meals for the entire family without appropriate cleaning.

### Misconceptions regarding LF and its vector

This study revealed poor knowledge and misconceptions regarding LF and its vectors. LF was referred to as the EVD (2014–2016), probably because of the severity of EVD in West Africa. Similar misconceptions regarding LF vectors were reported in Sierra Leone, where people mentioned shrews as the possible reservoir of the disease [16]. Bush mice (including *M*. *natalensis*) are hunted and consumed by children because they were not considered dangerous to human health. The beliefs and misconceptions that bush mice are not infectious remain a major indicator that more information regarding LF and its vector should be provided to these people. Although a previous study reported that people gained knowledge about LF in these villages through a rodent control intervention project [40], our study unveiled the limitation of the project on LF (i.e., the communication mechanism). The information was mainly provided through direct and brief talks with people during rodent sampling in their houses. Communication was mostly limited to participants and people found on spots during the sampling. Furthermore, some social ascription to “Sankaran” people (inhabitants of the Faranah region) with a reputation of practicing black magic may have prevented some of the project members from talking about the deadly disease during meetings with communities.

### Strengths and limitations

Our findings are part of a research on LF that has been ongoing since 2003 in this region. This facilitated the prolonged engagement with community members, which is the key to establishing quality and trustworthiness in qualitative research, especially with the credibility of the study [56].

However, information bias may exist in this type of study. Because adults were invited to talk about a sensitive issue related to the behavior of their children (consumption of rodents) in the village, and because the workers of the project of LF had urged them to ban their children from consuming mice, a social desirability bias may exist. Therefore, participant observations during hunting sessions and informal discussions with various community members facilitated the minimization of these biases by triangulating the data collected from these different sources. Finally, some people report that they do not know the disease to even receive more information on the disease.

Our anthropological analysis of this process allowed us to understand why and how children are in constant contact with mice, which may include the LASV reservoir (*M*. *natalensis*) beyond domestic limits. However, this study could not determine whether mice caught by children included *M*. *natalensis* that harbored the virus. Moreover, we did not explore the role of domestic animals, such as dogs in the transmission of LASV. Therefore, further epidemiological, virological, and ecological investigations are required to clearly identify the degree to which hunting practices constitute a risk of contracting LASV by children in this area.

The findings of this study revealed the factors that influence rodent hunting by children in these villages. The strong connection of these factors to the social life of community members places rodent hunting in the socialization process of children in these localities. Rodent hunting is a deeply rooted practice in childhood and adolescence [9,24,25,28,37]. The sociocultural aspect of this practice, coupled with poverty in settings with limited access to meat, renders it difficult to abandon the activity. These rodents are easy to catch and are almost exclusively consumed by children. Similar findings have been reported elsewhere, showing that people tend to consume more unhealthy food when available at minimal or no cost [57–60]. Hence, it is not relevant to vehicle messages such as “stop hunting.” Rather, it seems more relevant to develop some community-based strategies that could alleviate the risk of direct contact with rodents, especially mice, which is the LASV vector. Thus, safe hunting can be promoted by changing the target game, by educating children in schools and homes regarding health risks and thereby re-orient their hunting practice toward other rodents that do not harbor LASV and other pathogens and continue developing their identities as boys.

Given the importance of the sociocultural aspect of childhood hunting practices, it is relevant to define appropriate community strategies based on a collective “One Health” approach [61], considering the childhood sociocultural events and their implications to public health in rural communities. This may include conducting regular communication and awareness programs on LF and its vector in communities and schools, thereby encouraging and motivating parents to send their children to school and providing education at home through storytelling. Furthermore, the empowerment of parents with income-generating activities, such as vegetables and fruit farming, poultry, bee farming, and fish farming would develop alternative sources of meat for their children.

## Supporting information

S1 Table Excerpts from discussions on hunting places, techniques, cooking, sharing practices, and main causes (E: Excerpts, IDI: In-Depth Interview, FGD: Focus Group Discussion). (DOCX)
