# Correction: Hunting and consumption of rodents by children in the Lassa fever endemic area of Faranah, Guinea

**DOI:** 10.1371/journal.pntd.0011078

**Published:** 2023-01-24

**Authors:** Moussa Douno, Emmanuel Asampong, N’Faly Magassouba, Elisabeth Fichet-Calvet, Almudena Marí Sáez

The fifth author’s name is spelled incorrectly. The correct name is: Almudena Marí Sáez. The correct citation is: Douno M, Asampong E, Magassouba N, Fichet-Calvet E, Marí Sáez A (2021) Hunting and consumption of rodents by children in the Lassa fever endemic area of Faranah, Guinea. PLoS Negl Trop Dis 15(3): e0009212. https://doi.org/10.1371/journal.pntd.0009212
