# Supplementary material for: Hunting and consumption of rodents by children in the Lassa fever endemic area of Faranah, Guinea
Source: PLoS Negl Trop Dis. 2021 Mar 17;15(3):e0009212. doi: 10.1371/journal.pntd.0009212 (PMC7968712; doi:10.1371/journal.pntd.0009212)
Supplement: S1 Table — (DOCX) [file pntd.0009212.s001.docx]

**Hunting and consumption of rodents by children in the Lassa fever endemic area of Faranah, Guinea**

Short title: Children consuming rodents in Lassa fever endemic area of Guinea

Moussa Douno^1,2*^, Emmanuel Asampong^2^, N’Faly Magassouba^1^, Elisabeth Fichet-Calvet^3^, Marí Sáez Almudena^4^

^1^ Projet des Fièvres Hémorragiques en Guinée, Centre de Recherche en Virologie, Université de Conakry, Guinée

^2^ Department of Social and Behavioral Sciences, School of Public Health, University of Ghana, Legon, Accra, Ghana

^3^ Department of Virology, Bernhard Nocht Institute for Tropical Medicine, Hamburg, Germany

^4^ Center for International Health Protection, Robert Koch Institute, Berlin, Germany

*****[msokadouno@gmail.com](mailto:msokadouno@gmail.com)

**Table:** Excerpts from discussions on hunting places, techniques, cooking, sharing practices, hunting motivating factors and knowledge of LF (E: Excerpts, IDI: In-Depth Interview, FGD: Focus Group Discussion).

| **E1** | *"There is no burrow in these areas here that is not dug, there is none at all; rodent hunting is a widespread practice"* (IDI, 65-year-old man, former hunter). |
| --- | --- |
| **E2** | *"Children practice this hunting mainly because they are informed that their elders from previous generations were hunting rodents, which is what gives them the courage to do so; it has become a kind of custom, a generational culture, all children practice it at this age"* (IDI, 55-year-old man, community leader). |
| **E3** | *“… When we see a burrow, we bring in our hands to see if there are rat hairs. If it is a rat burrow, we find hair in, and that insures us that it is a rat burrow, so we dig it. The closer we get to the rat, we hear noise in it. But if it is a snake that is in a burrow, when you put your hand in it, it is the heat that you feel; then we realize that it is not a rat, we immediately remove hand and we move back away from it.* (FGD, 11-year-old unschooled boy). |
| **E4** | *“When we go to the bush, we search for burrows on the termite mounds; if we suspect the presence of a rat, we bring the dogs for smelling the burrows. If there is an animal in, the dog becomes excited and starts digging, then we dig too.* *If it comes out suddenly and climbs a tree, someone can climb after it and shake the tree’s branches. If we have a slingshot, he who is skillful knocks it and it goes down; if it tries to escape, dogs chase it and catch it; we then slaughter it* (FGD, 15-year-old pupil). |
| **E5** | *“It is dangerous to dig burrows without being accompanied by a dog; if you do not have a dog it is not safe to put your hand in a burrow. When we see a burrow, we incite the dog to feel the entrance, if the dog gets excited and digs, we also dig it, because it means that there is an animal. Contrary, if the dog moves back and gets away from the burrow, we move away from it too, because that means there is a snake in it”.* (FGD, 13-year-old unschooled boy). |
| **E6** | *“… we give a mouse to each child who comes with us and they go home; we too, we go home with the rest, we roast and eat with our little brothers, our little sisters and our friends.”* (FGD, 12-year-old unschooled boy). |
| **E7** | *“Sometimes, when they come from hunting, they take oil with me here and cook their meat here in my cooking pot. Sometimes, if their meat is a lot, they share it and everyone carries his part to his home for cooking and eat”* (IDI, 35 years old woman). |
| **E8** | *“We only eat meat of small animals from the bush. Other than that, if chicks die for example at home, or chickens, sheep, we can take them and skin them, we contribute among friends, each gives 500 Guinean francs (about 0.05$), we buy oil, Maggi cube, we roast and then eat”* (FGD, 10 years old pupil). |
| **E9** | *“Sometimes, they can bring back rats home to sell them to some people who say it is a medicine that treats high blood pressure; there are even some people from Faranah (the city) who buy them for the purpose of treating high blood pressure …”* (IDI, 55-year-old man, community leader). |
| **E10** | *“Children go for hunting because of meat, since their parents cannot afford to find it for them”* (IDI, 47 years old man, community leader). |
| **E11** | *"Hunting you're talking about has not started in our time, it is since the time of our great grandparents that the practice of hunting has started ... Even I who am speaking to you now, I practiced it when I was a child, so it is hard for children to stop it* (IDI, 55-years-old man, community leader). |
| **E12** | *"A boy should not sit like that without going for a walk in the bush to get something ..."* (FGD, 15-year-old unschooled boy). |
| **E13** | *“Bush is also a refuge place for us, because when we are there, we can do our childhood stubbornness including playing and hunting without being interrupted by parents. In the village, when we are stubborn, parents blame us but if we are in the bush, we are sheltered from them. We can spend good moments with friends by hunting and eating together”* (FGD 15-year-old pupil). |
| **E14** | *“Those who cannot do it are ‘‘women’’, they do not have the heart of a man; they are ‘‘fake men’’. When you come with your prey, they follow you to give them”* (FGD, 12-year-old unschooled boy). |
| **E15** | *“I have not heard of that disease yet. The disease because of which we were forbidden to eat bush meat, even the rat, we were not eating it, is Ebola; and that's over, so we can eat it safely now. Even bats, we were told not to eat it during Ebola. At that moment, my father had even forbidden me to go into the bush and warned me that he would kill my dog if I did not stop hunting. Finally, he killed my dog to prevent me from going for hunting. It was when it was declared that Ebola was over that we started hunting again”* (FGD, 13-year-old unschooled boy). |
| **E16** | *“I did not know this disease before until mouse elimination project arrived. They told us that this disease causes bleeding from the nose and all the openings of the man”* (IDI, 47 years old man,)*.* |
| **E17** | *“I do not know the signs of that disease. What has been said to me is that it can aggravate malaria in the body …”* (IDI, 65 years old man)*.* |
| **E18** | *“I heard through you who come to capture mice in our houses here that mouse can transmit a disease to human but I do not know exactly the type of disease”* (IDI, 42 years old women)*.* |
| **E19** | *“… Digging burrows is a risk, because by doing it, you do not know if it's a snake that is there or what, we just do it by "man's courage"* (FGD, 13 years old pupil, FGD-V1). |
| **E20** | *“Once, I went to Sansanko (a nearby village) and I found a case of snakebite. A group of children went for hunting; they dug a first burrow in which they captured a rat. Afterwards, they saw a second one that they began to dig. One of the children brought his hand into the burrow to check, unfortunately, there was a snake in it that immediately bit him on his hand. They tried to transport him to a nearby village to treat him, but unfortunately he died on the way behind our village here”* (IDI, 55 years old man, community leader). |
| **E21** | *“It is only God who protects children during hunting; myself, I was bitten twice by snake during my childhood, but I did not have a problem. At that time, the snakebites were not so serious, but now when a snake bites someone, it is only God who saves him”* (65-year old man, former hunter, IDI-V2). |
